# Persisting Cutaneous Pancreatic Fistula in a Patient With Necrotizing Pancreatitis: A Novel Approach of Transfistulous Histoacryl Occlusion

**DOI:** 10.14309/crj.0000000000001456

**Published:** 2024-08-22

**Authors:** Julian Cardinal von Widdern, Franz Stangl, Walter A. Wohlgemuth, Richard Brill, Jörg Kleeff, Jonas Rosendahl

**Affiliations:** 1 Department for Internal Medicine I (Gastroenterology, Pulmonology), University Hospital Halle (Saale), Halle, Germany; 2 Department for Diagnostic and Interventional Radiology, University Hospital Halle (Saale), Halle, Germany; 3 Department of Diagnostic and Interventional Neuroradiology, University Hospital Augsburg, Augsburg, Germany; 4 Department of Visceral, Vascular and Endocrine Surgery, University Hospital Halle (Saale), Halle, Germany

**Keywords:** acute pancreatitis, pancreatic fistula, interventional treatment

## Abstract

Necrotizing pancreatitis with superinfection of necrotic tissue is associated with a high rate of complications and mortality. The step-up approach is a well-established treatment strategy for necrotizing pancreatitis, emphasizing minimally invasive and endoscopic interventions before considering surgical options. Minimally invasive strategies often involve percutaneous drainage of collections, which carries the risk of persisting cutaneous pancreatic fistulas. Since there is currently no guidance for managing this scenario, we present a novel treatment approach that utilized tissue glue to occlude a persisting and clinically compromising percutaneous fistula. In addition, we summarize the current knowledge in the treatment of percutaneous pancreatic fistulas and provide a potential therapeutic algorithm for further evaluation.

## INTRODUCTION

Acute necrotizing pancreatitis continues to pose a significant mortality risk despite advancements in interventional therapy, particularly among patients with infected necrosis.^1^ The step-up approach is a well-established concept in the treatment of necrotizing acute pancreatitis. Conservative, endoscopic, or interventional approaches have proven effective in delaying or even avoiding surgical therapy. Moreover, they have reduced major complications including fatalities.^2^ Meanwhile, endoscopic transgastric necrosectomy has been shown to be equally effective as the surgical minimally invasive step-up approach.^3^ Although transgastric necrosectomy can be successful without additional percutaneous drainage, in several patients, the size and localization of infected necrosis necessitate such interventions. Drainage procedures can lead to fistulas in up to 15% of patients, necessitating interdisciplinary treatment concepts.^4^ Although the majority of these fistulas appear to close spontaneously, persistent ones pose a challenge often jeopardizing patient recovery. Given the recent guideline recommendations favoring minimally invasive treatments, including percutaneous drainage in specific situations, the likelihood of pancreatic fistulas is expected to rise, underscoring the urgent need for therapeutic algorithms in this scenario.^5^

## CASE REPORT

This is the case of a 39-year-old healthy man who developed severe acute necrotizing pancreatitis of unknown etiology. The patient had no history of alcohol consumption, endosonography ruled out choledocholithiasis, and serum triglycerides and calcium levels were normal. The course was complicated by acute respiratory distress syndrome, necessitating mechanical ventilation and extracorporeal membrane oxygenation in the first weeks. Subsequently, there was a renewed increase in inflammatory markers, and a computed tomography (CT) scan revealed extensive retroperitoneal walled-off necrosis with entrapped gas and wall enhancement as signs of infection. The necrotic cavity was drained transgastrically under endoscopic ultrasound guidance and required several sessions of transgastric necrosectomy with endoscopic snares. Owing to the extensive dimensions of the necrosis formation extending into the pelvis (19 × 14 × 11 cm) and individual sections not accessible transgastrically, a percutaneous large lumen drainage (36 French) was additionally surgically installed. After clearance of the necrotic material, the transcutaneous drainages were gradually withdrawn and finally removed. A CT scan confirmed the complete resolution of the necrotic cavity. After more than 4 months in the hospital, with several weeks under intensive care, the patient was transferred to a rehabilitation facility, from where he was discharged home.

**Figure 1.**
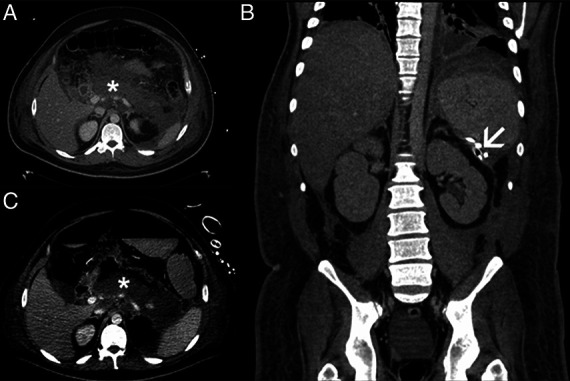
Extensive peripancreatic and retroperitoneal necrosis indicated by an asterisk (*) in the computed tomography of the patient (A, B). Retroperitoneal necrotic areas were treated with the installation of a transcutaneous drainage extending to the splenic hilum (as shown by the arrowhead) (C).

In the outpatient clinic, the patient presented without signs of endocrine or exocrine insufficiency and reported no complaints in the following months. However, after 5 months, a fluid collection (measuring 5 × 3 cm) appeared in the left flank along with a cutaneous fistula at the site where the percutaneous drainage had been previously installed (Figure 1). Surgical closure was unsuccessful, and a magnetic resonance imaging (MRI) revealed a connection between the subcutaneous fluid collection and the area of the suspected remnant pancreatic tail. Aspiration of the fluid revealed high levels of amylase (>50 μkat/L [The test is considered positive for a pancreatic fistula if the amylase activity in the secretion is measured to be 3 times higher than in the serum. In this patient, the serum amylase activity was 0.35 μkat/L. The reference range in serum is < 1.67 μkat/L.]) and no signs of infection. A residual pancreatic duct was observed only in the head of the pancreas, and no additional pancreatic tissue could be visualized on MRI. However, due to the course of the fistula tract and the detection of amylase in the secretion, we had to assume that there was minimal remaining pancreatic tissue in the area of the former pancreatic tail. Consequently, no endoscopic options for draining the percutaneous fistula through an intervention on the main pancreatic duct were available. Six months later, superinfection of the fluid collection and the surrounding subcutaneous tissue occurred. The patient presented with fever, elevated C-reactive protein, and purulent secretion from the fistula opening. Surgical partial excision of the fistula and installation of irrigation drainage were performed. The drainage was eventually removed after 2 months when the secretion ceased spontaneously and sonography confirmed resolution of the collection. Symptoms and fluid collection recurred after 1 month, and CT confirmed an extension of the formation to the area of the suspected remnant pancreatic tail and the hilum of the spleen. After interdisciplinary discussion, we opted for a minimally invasive radiological approach, as both surgical (due to potential bleeding complications because of portosplenic collaterals) and endoscopic (no pancreatic duct structure found in the pancreatic tail) strategies were deemed unsuitable.

**Figure 2.**
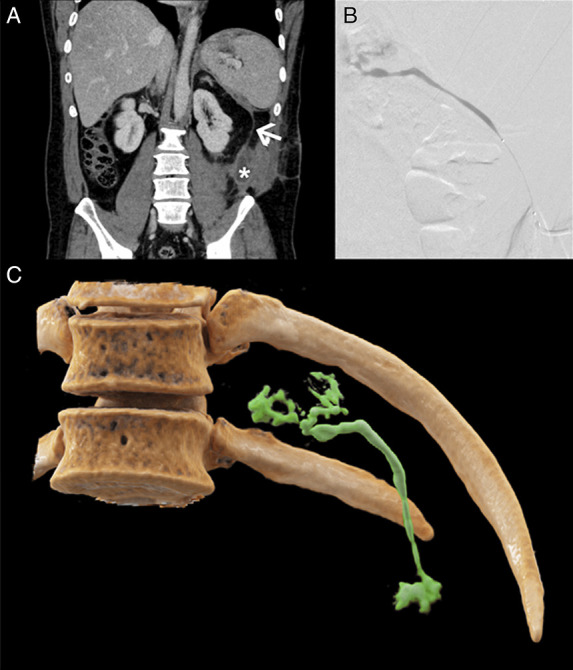
Computed tomography images of the patient depict subcutaneous fluid collection (indicated by asterisk [*]) caused by a pancreatic fistula in the area of the former percutaneous drainage (as shown by the arrowhead) (A). During the intervention, complete contrasting of the formation was achieved after injection of contrast medium and a 1:4 mixture of Histoacryl and lipiodol were installed (B). A 3D-reconstruction of the postinterventional result is shown in (C).

Here, the subcutaneous fluid collection was punctured under sonography guidance, and a 6-French lock was inserted. The fistula was probed with a Synchro 10 wire, and an Excelsior SL-10 Microcatheter (Stryker Neurovascular, Fremont, CA) was placed in the area of the remnant pancreatic tail. Contrast injection visualized the remnant pancreatic tail area and the fistula (Figure 2). To occlude the fistula, a mixture of 1 mL Histoacryl (N-butyl-2-cyanoacrylate) and 4 mL lipiodol was injected while retrieving the catheter. The subcutaneous fluid collection was aspirated during the removal of the 6-French lock. After the procedure, the patient developed clinical (abdominal pain) and laboratory signs (elevated lipase and C-reactive protein) of acute pancreatitis, which resolved after 2 days. The patient was discharged and has shown no signs of recurrence of the percutaneous fistula since then. In the meantime, pancreatic exocrine and endocrine insufficiencies have been diagnosed and are currently treated with pancreatic enzyme replacement therapy, metformin, and insulin (3 months after the procedure). The entire follow-up period now spans 4 years since the intervention, during which the patient has experienced no complications related to the procedure.

## DISCUSSION

**Figure 3.**
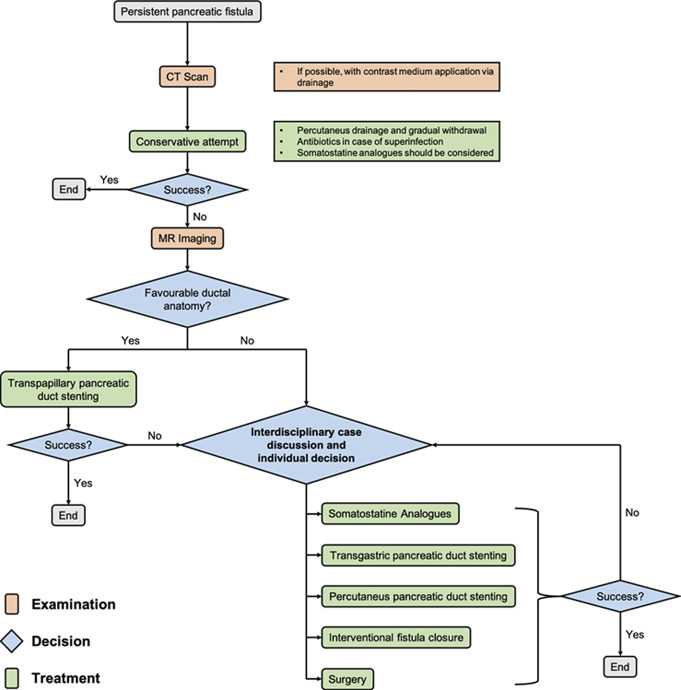
Proposal for structuring diagnostic and therapeutic measures for the treatment of pancreatic fistulas. CT, computed tomography; MR imaging, magnetic resonance imaging.

Changing treatment strategies for necrotizing pancreatitis with novel consecutive complications poses a significant challenge for physicians in effectively managing such scenarios. In cases of necrotizing pancreatitis, percutaneous catheter drainage followed by endoscopic interventions is associated with substantial long-term morbidity rates, reaching of up to 23%.^6^ The approach to maintaining the access route (eg, full-covered metal stents; diameter of the percutaneous drainage) likely influences subsequent complications. Common periprocedural complications reported include bleeding, pneumoperitoneum, peritonitis, and aspiration pneumonia.^6^ Moreover, pancreatico-cutaneous or pancreatico-enteric fistulas are prevalent complications of percutaneous drainage procedures, with a frequency exceeding 50%, as summarized in a recent meta-analysis.^4^ These data are supported by a monocentric retrospective analysis, which revealed enteric communication rates of 8.9% and bleeding rates of 7.3% in over 1,000 interventions.^7^ In the majority of cases, complications are self-limiting, or conservative approaches such as catheter withdrawal have been successful, demonstrating the capability of specialized centers to manage the situation effectively. So far, no universally agreed-upon therapeutic algorithms are available, as highlighted by the case presented here. Experience-based data suggest conservative management of pancreatico-enteric fistulas when clinically feasible. Following a CT scan with concomitant contrast medium application through the catheter, a stepwise withdrawal strategy is suggested, depending on clinical signs such as peritonitis and drain output.^7^ In the light of the increasing significance of percutaneous pancreatic fistulas following therapeutic interventions, an attempt to streamline therapeutic approaches is depicted in Figure 3.

MRI imaging holds promise in delineating interventional options, which may include pancreatic duct stenting through feasible access routes (transpapillary, transgastric, percutaneous) to address pancreatic duct leaks or ruptures.^8,9^ In cases where interventional options are limited, a conservative approach involving somatostatin analogs (eg, 100–200 µg octreotide every 8 hours) could be considered.^10^ The aim of this approach is to reduce pancreatic secretion, potentially facilitating self-induced fistula healing. If these conservative measures prove ineffective, surgical intervention should be considered. However, surgical procedures are often complicated by varicose bypass circuits resulting from thrombosed splanchnic veins. In rare instances, such as the 1 illustrated here, occlusion of the persistent fistula by radiological intervention offers a minimally invasive therapeutic option. Histoacryl has been successfully used for many years in the endoscopic treatment of gastric varices. A case series suggests that it can be used safely and effectively for the endoscopic closure of pancreatic fistulas.^11^ In addition, there are few case reports in the literature where Histoacryl has been used for the percutaneous closure of fistulas, such as postoperative pancreatic fistulas or bilio-cutaneous fistulas.^12,13^

This case underscores the importance for close interdisciplinary collaboration in managing complications of acute pancreatitis. There is an urgent need for prospective studies that (i) investigate the general treatment strategies of pancreatic fistulas and (ii) examine the safety and effectiveness of radiological-interventional fistula closure as presented here.

## DISCLOSURES

Author contributions: J. Cardinal von Widdern: writing the manuscript, treatment of the patient, revision. F. Stangl: revision of the manuscript, treatment of the patient. W. Wohlgemuth, R. Brill, J. Kleef: revision of the manuscript. J. Rosendahl: conceptualization, treatment of the patient, revision of the manuscript. J. Cardinal von Widdern is the article guarantor.

Acknowledgments: We thank the patient for allowing us to publish clinical data of the case.

Financial disclosure: None to report.

Informed consent was obtained for this case report.
